# Microarrays Reveal Early Transcriptional Events during the Termination of Larval Diapause in Natural Populations of the Mosquito, *Wyeomyia smithii*

**DOI:** 10.1371/journal.pone.0009574

**Published:** 2010-03-05

**Authors:** Kevin J. Emerson, William E. Bradshaw, Christina M. Holzapfel

**Affiliations:** Center for Ecology and Evolutionary Biology, University of Oregon, Eugene, Oregon, United States of America; University of Missouri-Kansas City, United States of America

## Abstract

**Background:**

The mosquito *Wyeomyia smithii* overwinters in a larval diapause that is initiated, maintained and terminated by day length (photoperiod). We use a forward genetic approach to investigate transcriptional events involved in the termination of diapause following exposure to long-days.

**Methods/Principal Findings:**

We incorporate a novel approach that compares two populations that differentially respond to a single day length. We identify 30 transcripts associated with differential response to day length. Most genes with a previously annotated function are consistent with their playing a role in the termination of diapause, in downstream developmental events, or in the transition from potentially oxygen-poor to oxygen-rich environments. One gene emerges from three separate forward genetic screens as a leading candidate for a gene contributing to the photoperiodic timing mechanism itself (photoperiodic switch). We name this gene *photoperiodic response gene 1* (*ppdrg1*). *WsPpdrg1* is up-regulated under long-day response conditions, is located under a QTL for critical photoperiod and is associated with critical photoperiod after 25 generations of recombination from a cross between extreme phenotypes.

**Conclusions:**

Three independent forward genetic approaches identify *WsPpdrg1* as a gene either involved in the photoperiodic switch mechanism or very tightly linked to a gene that is. We conclude that continued forward genetic approaches will be central to understanding not only the molecular basis of photoperiodism and diapause, but also the evolutionary potential of temperate and polar animal populations when confronted with rapid climate change.

## Introduction

At temperate latitudes, no life cycle is complete without the means to exploit the favorable season, to avoid or mitigate the unfavorable season, and to switch from one life style to the other in a timely manner. A diversity of temperate arthropods reduce the negative effects of winter by entering a state of hibernal dormancy (diapause, see Text S1 for a glossary of terms). At temperate and polar latitudes, most arthropods use the length of day, or photoperiod, to initiate and, in some cases, maintain and terminate diapause [1]–[5]. The timing of the transition between active development and diapause at the appropriate time of year in seasonal environments is central to maintaining fitness in natural populations [6], [7]. Over geographic gradients, winter arrives earlier at more northern or higher elevation localities and later at more southern or lower elevation localities. Concomitantly, the day length used to switch between active development and diapause (critical photoperiod) in the late summer or fall increases with latitude and altitude and is closely correlated with the length of the local growing season [7]–[9]. Critical photoperiod regulating the initiation or termination of diapause usually has a high heritability, responds rapidly to selection in the laboratory, and has shown rapid evolution (genetic change) in response to recent rapid climate change [10]–[13].

**Figure 1:**
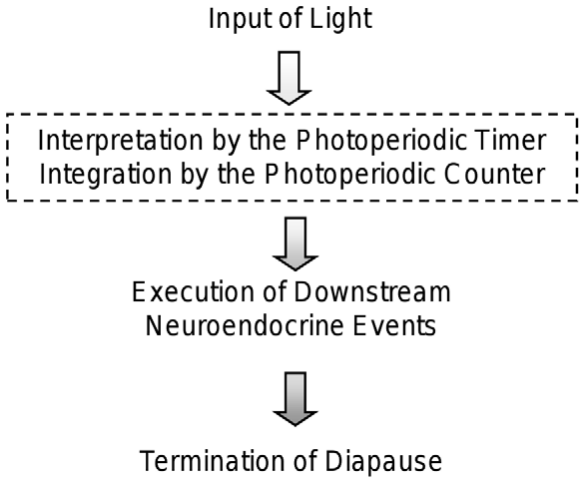
Schematic representation of the events leading to the termination of diapause. Day length is recognized and interpreted as short or long by the photoperiodic timer; the photoperiodic counter integrates day-length information and triggers a photoperiodic response when the threshold number of long days has been exceeded. In combination, the photoperiodic timer and counter act as a switch (dashed box) executing a cascade of neuroendocrine events that ultimately results in the termination of diapause and the resumption of development.

The use of photoperiod for the initiation or termination of diapause is a complex physiological process that involves (1) the input of light, (2) a photoperiodic switch mechanism that is comprised of a timer that assesses the length of day or night and a counter that keeps track of the number of inductive cycles and triggers the photoperiodic response when the threshold number of long or short days has been exceeded, (3) a neuroendocrine output signal, and (4) overt signs of diapause or development (Figure 1) [14]–[16]. More generally, photoperiodic response consists of an input of light that is recognized as a long or short day length by the photoperiodic timer, integrated by the photoperiodic counter, and executed through the neuroendocrine system to control seasonal events such as diapause.

Herein, we use the termination of diapause to investigate the photoperiodic switching mechanism. Usually, the termination of diapause is scored by an overt developmental event such as hatching, molting to the next stage of development, metamorphosis or reproductive activity [2], [15]. However, many genetic and physiological events take place between the input of light and overt signs of resumed development (i.e. molting). Using a cDNA microarray and a novel experimental approach to assess response to day length, we reveal the transcriptional regulation of genes involved in the early stages of post-diapause morphogenesis and metabolism and propose one particular gene as a strong candidate for involvement in the photoperiodic switch mechanism itself.

As a consequence of wide acceptance of Bünning's [17] hypothesis that daily circadian rhythms form the necessary, causal basis of the seasonal photoperiodic switch, candidate gene-based studies of the photoperiodic switch have emphasized known circadian rhythm genes [18]–[23]. These studies have led to a greater understanding of these genes in relation to their role in the circadian clock and diapause, but have shed little light into the genetic basis of the photoperiodic mechanism [14], [24], [25]. Forward genetic approaches, unbiased by historical hypotheses, need to be incorporated into studies of photoperiodism and diapause [14], [24]–[26]. Herein, we take such an approach, using microarrays and association analysis to scan for genes involved primarily in the photoperiodic switch and secondarily in the termination of larval diapause in the mosquito *Wyeomyia smithii*.

*Wyeomyia smithii* completes its pre-adult development exclusively within the water-filled leaves of the purple pitcher-plant, *Sarracenia purpurea*. Throughout its range in eastern North America, *W. smithii* undergoes an hibernal larval diapause (as III or IV instar larvae) that is initiated or maintained by short days, and is averted or terminated by long days [27]. The photoperiodic timer is measured by the critical photoperiod, i.e., the day length used to switch between active development and dormancy; the photoperiodic counter is measured by the depth (or intensity) of diapause, i.e., the number of long days required to terminate diapause. Both the critical photoperiod and the depth of diapause increase with latitude and altitude of population origin [10], [27].

The precise timing of diapause termination in populations of *W. smithii* has been estimated using “pulse-chase” experiments [28]. In these experiments, all individuals within a population are first synchronized into diapause by rearing them on short days. In separate experiments (with separate individuals), cohorts of diapausing larvae are exposed to 1, 2, 3 or more long-days and then returned to short-days. After a given number of long-days individuals become irreversibly committed to diapause termination (as measured by future development after a return to short-days) even though no morphological differences can be seen at the time the go/no-go ‘decision’ is made. This ability to synchronize thousands of animals in diapause, and to measure explicitly the timing of the physiological transition between diapause and diapause termination makes diapause termination in *W. smithii* a unique model system for studies of photoperiodism.

We use the power of natural geographic variation in properties of the photoperiodic time measurement system and diapause termination to determine differential gene expression in response to day length.

### Experimental Approach and Design

**Figure 2:**
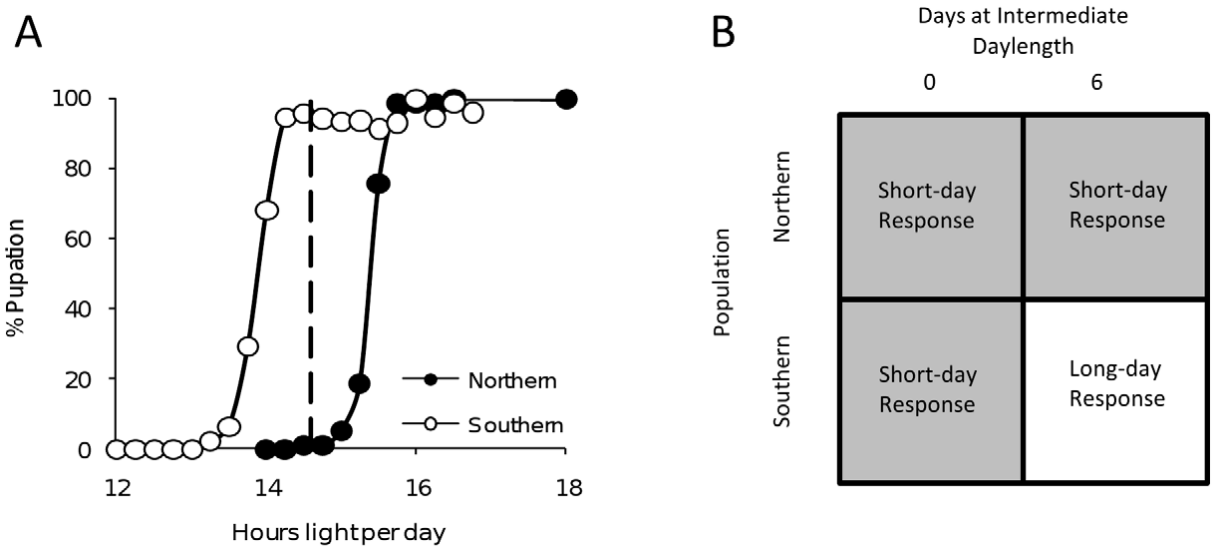
Experimental design for microarray experiments. (A) Photoperiodic response curves of a northern population (Ontario, Canada; 46°N, 150 m elevation) and a southern population (North Carolina, USA; 35°N, 900 m elevation). The dashed line shows 14.6 hours of light per day, perceived by the southern population as a diapause-terminating long day and by the northern population as a diapause-maintaining short day. This intermediate day length was used in the experiments described herein. (B) Microarray experimental design. Northern and southern individuals were transferred from short days (L∶D = 10∶14) to the experimental day length (L∶D = 14.6∶9.4) on day 0. RNA was extracted from larvae 10 h after dawn on days 0 and 6. On day 0, none of the animals had seen any long days. On day 6, larvae from the southern but not the northern population had interpreted L∶D = 14.6∶9.4 as long days, at which time 50% of the larvae had terminated diapause and were committed to renewed development even though there were no overt signs of development [28]. The cells in the figure show the four experimental treatments and the physiological interpretation of those treatments (short-day response–diapause maintenance; long-day response–diapause termination).

To detect expression of genes involved in the photoperiodic switch and initial stages in the termination of diapause, we use a cDNA expression microarray to determine differential gene expression between two populations that respond differently to a single day length (Figure 2A). The populations represent a geographic extreme of populations that diapause in the same (III) instar. One population interprets this day length as a diapause-terminating long day, whereas the other population interprets this same day length as a diapause-maintaining short day. On day zero of the experiment, mosquitoes are transferred from short days (Light∶Dark = L∶D = 10∶14) to the experimental L∶D cycle (L∶D = 14.6∶9.4) at 23°C. The experimental L∶D cycle is interpreted as a long day by <2% of animals in the northern population (Ontario, Canada) and by >98% of animals in the southern population (North Carolina, USA) (Figure 2A). Transitions between L∶D cycles maintained a constant time of dawn. RNA samples (six replicates of 50 whole larvae per day per population) were collected at 10 h after lights-on on days zero and six (Figure 2B). Since dawn was synchronized, no long-days have been recognized by either population by 10 h after dawn on day zero, and these samples act as a control for evolved differences in gene expression between the two populations that are unrelated to photoperiodic response. By 10 h after dawn on day six, more than 50% of individuals within the southern population have terminated diapause, even though there are no overt signs of development in these larvae [28]. At the same time, none of the individuals from the northern population have received any signals that they interpret as diapause-terminating long-days. This design controls for (1) differences in gene expression between the northern and the southern population unrelated to photoperiodic response through differences between populations on day zero and (2) differences in gene expression due to day length but not involved in photoperiodic response through differences in gene expression within the northern population between the samples on days zero and six. Differential expression of genes due to the photoperiodic switch and post-diapause morphogenesis is then assessed by using linear models to test for transcripts that display a significant interaction between population and day (0 or 6) of experiment. In particular, we focus on those genes whose expression is consistent with the particular type of interaction in which the genes are differentially expressed in the one long-day response condition (Figure 2B) relative to the three short-day response conditions.

We noted that one candidate gene resides under a QTL for the evolution of photoperiodic response [29]. We tested whether this gene was significantly associated with photoperiodic response in an F_26_ hybrid generation between a southern (short critical photoperiod) and a northern (long critical photoperiod) population by genotyping a total of 31 short and 28 long critical photoperiod individuals at this locus.

## Results

### Microarrays

**Figure 3:**
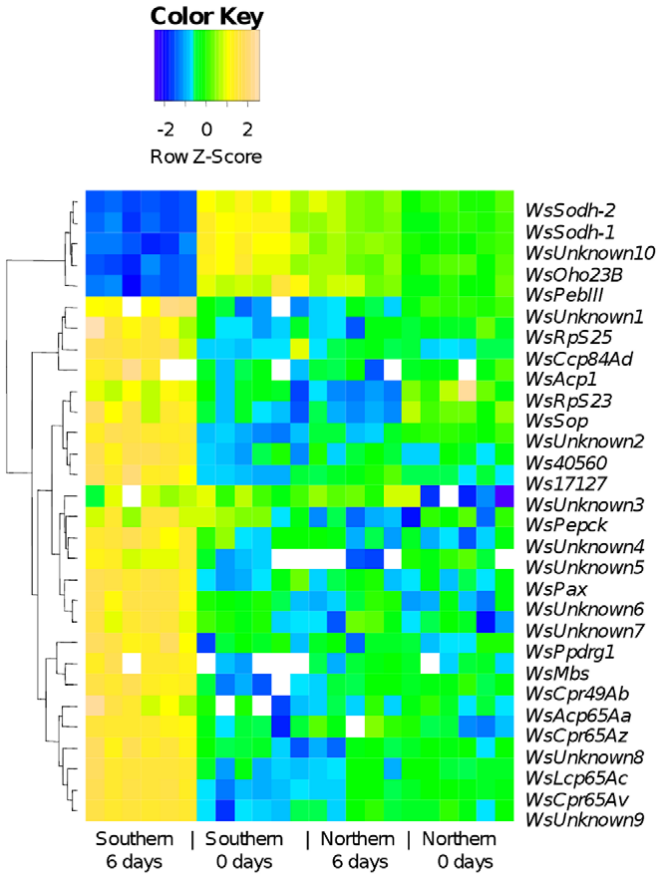
Gene expression that is regulated by differential physiological response to day length. Cell color denotes expression level standardized within genes across all experimental treatments relative to a reference sample; white cells represent missing data. The four different treatments from Figure 2B are represented along the bottom of the figure. Note that all of the genes presented here have a different pattern of expression in the single treatment generating a long-day response (Southern day 6) compared with the other three treatments.

**Figure 4:**
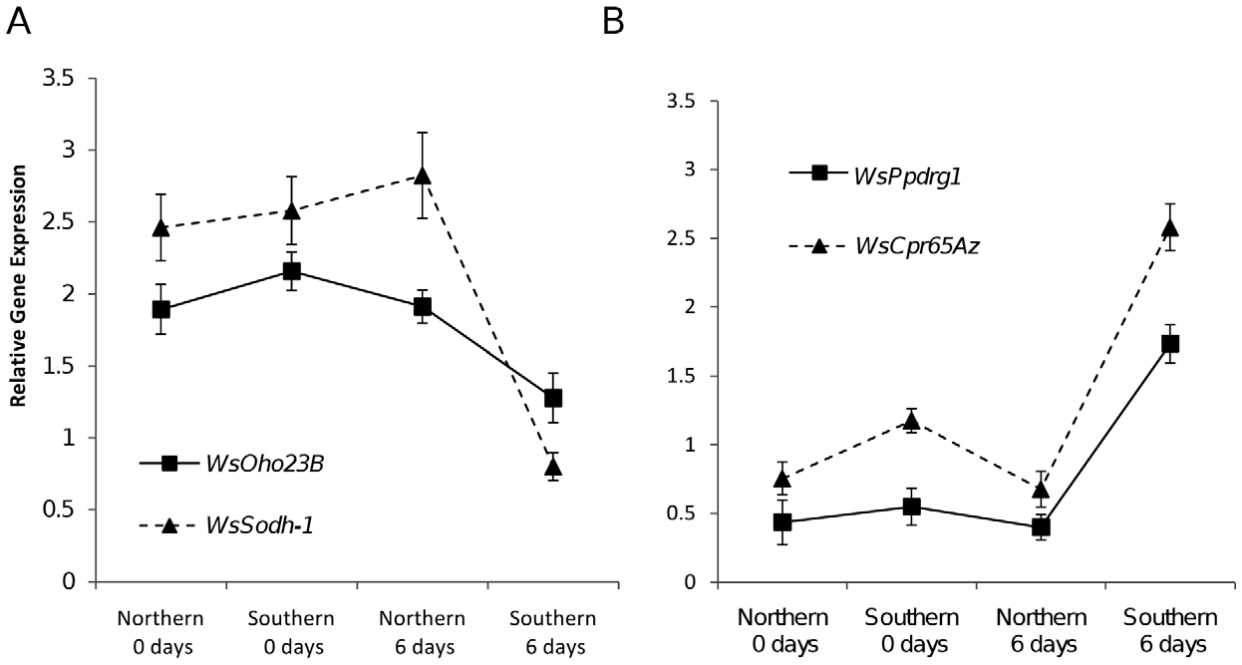
qRT-PCR validation of microarray results. Gene expression of four genes isolated from a microarray screen for candidate genes involved in the photoperiodic termination of diapause, relative to the reference gene *Rp49* [18]. (A) Two genes that are down-regulated under long-day response treatments, (B) Two genes that are up-regulated under long-day response treatments. Error bars represent one standard error.

After correction for multiple testing using a Benjamini-Hochberg False Discovery Rate of 0.01 [30], 188 elements on the microarray showed a significant population x day (0 or 6) interaction, of which 62 showed expression patterns consistent with differential expression under long-day response conditions. These 62 elements corresponded to 30 transcripts that become candidate genes for the photoperiodic switch, for diapause termination, or for post-diapause morphogenesis (Table 1). Five transcripts were significantly down-regulated and 25 transcripts were significantly up-regulated in the long-day response treatment (Figure 3). Ten of the genes are orphan genes in *W. smithii*, having no significantly similar sequences found in *D. melanogaster, Aedes aegypti*, or *Anopheles gambiae*. One transcript, that we name *photoperiodic response gene 1* (*ppdrg1*), is a member of a family of genes including the RetininC superfamily domain (Pfam04527/IPR007614) (Figure S1) [31]. *WsPpdrg1* is significantly upregulated under long-day response conditions. Expression patterns of two down-regulated genes (*WsSodh-1* and *WsOho23B*) and two up-regulated genes (*WsPpdrg1*) and *WsCpr65Az*) were confirmed by quantitative real-time PCR (Figure 4) using gene-specific primers (Table S1). *WsSodh-1* and *WsOho23B* were both significantly down-regulated in the long-day treatment relative to the other treatments (*WsSodh-1*: *F*_1,8_ = 51.42, *P*<0.001, *WsOho23B*: *F*_1,8_ = 16.63, *P* = 0.002). *WsPpdrg1* and *WsCpr65Az* were both significantly up-regulated in the long-day treatment relative to the other treatments (*WsPdrg1*: *F*_1,8_ = 77.64, *P*<0.001, *WsCpr65Az*: *F*_1,8_ = 77.83, *P*<0.001).

**Table 1:** List of genes shown in Figure 3.

		*Drosophila*		
	Genbank	Ortholog		Adjusted
Gene Symbol^1^	Accession #	Flybase ID	Gene Name	P value^2^
***Genes with lower expression in long-day response treatment***				
*WsSodh-1*	GW420671	FBgn0024289	Sorbitol dehydrogenase 1	2.97E-05
*WsPebIII*	GW420667	FBgn0011695	Ejaculatory bulb protein III	5.65E-05
*WsSodh-2*	GW420672	FBgn0022359	Sorbitol dehydrogenase 2	5.78E-05
*WsOho23B*	GW420665	FBgn0015521	Overgrown hematopoietic organs at 23B	6.31E-05
*WsUnknown10*	GW420675		Ws Unknown 10	1.36E-04
***Genes with higher expression in long-day repsonse treatment***				
*WsUnknown2*	GW420676		Ws Unknown 2	3.55E-06
*WsPpdrg1*	GU320742	FBgn0036600	Photoperiodic response gene 1	7.68E-06
*WsUnknown9*	GW420683		Ws Unknown 9	1.02E-05
*WsPax*	GW420666	FBgn0041789	Paxillan	2.06E-05
*WsCpr49Ab*	GW420660	FBgn0050042	Cuticular protein 49ab	2.10E-05
*WsUnknown8*	GW420682		Ws Unknown 8	2.38E-05
*WsLcp65Ac*	GW420663	FBgn0020642	Larval cuticle protein 65ac	2.53E-05
*WsCpr65Az*	GW420662	FBgn0035686	Cuticular protein 65az	2.90E-05
*WsUnknown3*	GW420677		Ws Unknown 3	2.97E-05
*WsRpS23*	GW420669	FBgn0033912	Ribosomal protein S 23	3.14E-05
*WsUnknown7*	GW420681		Ws Unknown 7	4.06E-05
*WsPepck*	GW420668	FBgn0003067	Phosphoenolpyrovate carboxylkinase	4.26E-05
*WsCpr65Av*	GW420661	FBgn0052405	Cuticular protein 65av	4.35E-05
*WsUnknown4*	GW420678		Ws Unknown 4	4.44E-05
*WsCcp84Ad*	GW420659	FBgn0004780	Cuticular protein 84ad	6.06E-05
*Ws17127*	GW420655	FBgn0032299	Ws17127	6.19E-05
*Ws40560*	GW420656	FBgn0085743	Non protein coding gene	6.27E-05
*WsAcp1*	GW420657	FBgn0014454	Adult cuticle protein 1	9.00E-05
*WsSop*	GW420673	FBgn0004867	String of Pearls	9.09E-05
*WsAcp65Aa*	GW420658	FBgn0020765	Adult cuticle protein 65A	9.22E-05
*WsMbs*	GW420664	FBgn0002690	Myosin binding subunit	1.04E-04
*WsUnknown1*	GW420674		Ws Unknown 1	1.15E-04
*WsUnknown5*	GW420679		Ws Unknown 5	1.36E-04
*WsRpS25*	GW420670	FBgn0086472	Ribosomal protein S 25	1.36E-04

1 Gene names were assigned the names from the highest scoring *blastx* hit (E<0.001) from *D. melanogaster*, preceded by “*Ws*”. Where there were no significant blast results, genes were named *WsUnknown* followed by a number.

2 Adjusted P values for the interaction term between day and population from the linear model analysis of the gene expression data.

### Association Analysis

One gene, *WsPpdrg1*, occurs under a major QTL for critical photoperiod [29]. We therefore tested whether this gene was still associated with critical photoperiod after 25 generations of recombination in a cross between a northern (Alberta) and southern (Florida) population (See methods). Southern populations diapause in the fourth larval instar, northern populations diapause in the third larval instar, and hybrids are polymorphic [27]. We therefore tested for an association between critical photoperiods and southern vs. northern allele frequencies in third and fourth instars separately. There was a positive association between critical photoperiod and frequency of the northern allele in *WsPpdrg1* for both third instar (χ^2^ = 9.38, d.f. = 1, *P* = 0.002) and fourth instar (χ^2^ = 10.30, d.f. = 1, *P* = 0.001) diapausing larvae (Table S2).

## Discussion

Our long-range goal is to identify genes specifically involved in the photoperiodic switch that ultimately leads to the termination of diapause and the initiation of post-diapause morphogenesis. As an initial step towards this goal, we exploit two properties of photoperiodic response in *Wyeomyia smithii*. First, we are able to expose two populations to a single day length that the northern population interprets as a diapause-maintaining short day and the southern population interprets as a diapause-terminating long day (Figure 2A). Second, we know from pulse-chase experiments [29] that after six long days, at least 50% of a larval cohort from the southern population has terminated diapause and is irrevocably committed to resumed development. At this point in time, there are no overt signs of post-diapause morphogenesis and we use a forward genetic approach with an expression microarray to reveal internal genetic and physiological processes leading to the termination of diapause. We are fully aware that most of the genes we identify are likely to be developmental or metabolic genes downstream from the photoperiodic switch mechanism. Where we are able to identify orthologous genes in *Drosophila melanogaster*, we assume that the ortholog in *W. smithii* serves the same function as that annotated in *D. melanogaster*. However, since at this time there are no known genes comprising the photoperiodic switch in natural populations of any animal, we fully expect that candidate loci in *W. smithii* might have no annotated ortholog in *D. melanogaster*. Below we consider differentially expressed genes in *W. smithii* that, by virtue of their annotated function in *D. melanogaster*, are involved in post-diapause morphogeneis, or in the transition from potentially oxygen-poor winter diapause environments to oxygen-rich spring environments conducive to active development. We then focus on a candidate locus for involvement in the photoperiodic switch mechanism.

### Post-Diapause Morphogenesis

*Paxillin* (*Pax*) in *D. melanogaster* is involved in cytoskeletal anchoring at the plasma membrane and in cell-cell adhesion during growth [32]. Up-regulation of *WsPax* in response to long days in *W. smithii* is consistent with its role in growth in *D. melanogaster*. *Myosin binding subunit* (*Mbs*) in *D. melanogaster* is involved in multiple developmental events including regulation of photoreceptor development in the compound eye [33]. In *W. smithii*, the pigmented adult eye is easily visible in fourth instar larvae so that up-regulation of *WsMbs* upon the termination of third instar diapause and the initiation of fourth instar development is likely an indicator of adult eye differentiation. Differential expression of cuticle genes has been found in pea aphids under different day-length conditions [34] where the modification of the cuticle structure may act as a response to photoperiod [35]. This observation, combined with the fact that diapause termination in *W. smithii* is associated with the molt from third to fourth instar larvae is consistent with the up-regulation of genes coding for cuticle proteins in response to long days.

In *Drosophila melanogaster*, *overgrown hematopoietic organs at 23B* (*oho23B*) codes for a ribosomal protein whose under-expression enhances imaginal disc overgrowth [36]. In the Mediterranean fruit fly, *Ceratitis capitata*, *oho23B* expression decreases as larval development proceeds, reaching a minimum in the prepupal stage [37]. Several ribosomal protein genes have been shown to be up-regulated during diapause in the northern house mosquito *Culex pipiens* [38]. The down-regulation of the expression of *WsOho23B* and the up-regulation of other ribosomal protein genes in *W. smithii* in response to long days is therefore consistent with other studies of insect development and diapause.

### Changes in Expression of Metabolic Genes

Long days promoting the termination of diapause elicit transcriptional profiles consistent with a transition from potentially oxygen poor to oxygen rich environments. *Wyeomyia smithii* overwinter as diapausing larvae within the water-filled leaves of pitcher plants. Both in high altitude southern and in northern populations, pitcher-plant leaves are routinely covered with snow and either frozen to the base of the leaf or partially frozen with a small aqueous portion containing crowded larvae and detritus at its base. Under these conditions, there is little or no opportunity for oxygen exchange with the atmosphere and diapausing larvae are likely encountering hypoxic conditions. Under warm spring and summer conditions when larvae have terminated diapause and are actively developing, oxygen generated by photosynthesis is infused into the basal portion of the leaves [39]. Hence, the transition from winter diapause to vernal development is accompanied by a transition from potentially oxygen poor to oxygen rich environments. Differences in the transcription of metabolic genes reflect these environmental transitions.

*Phosphoenolpyruvate carboxykinase* (*Pepck*) catalyzes the conversion of oxaloacetate to phosphoenolpyruvate, the rate-limiting step in the metabolic pathway that produces glucose from noncarbohydrate precursors [40]. Since hypoxia can lead to anaerobic respiration and the buildup of lactate, acetate and alanine in *D. melanogaster* [41], we propose that the up-regulation of *WsPepck* relates to the transition from an oxygen poor to an oxygen rich environment.

*Sorbitol dehydrogense* (*Sodh*) is a two gene system (*Sodh-1 and Sodh-2*) composed of paralogous genes that appear in tandem in insect genomes [42] and have a highly conserved sequence (>90% within both *W. smithii* and *D. melanogaster*). In *W. smithii*, both copies of *WsSodh* are highly expressed in larval diapause, and are down-regulated after exposure to diapause-terminating long days (Figure 3). *Sodh* also shows diapause-related expression changes in other insects: *Sodh* is expressed late in the embryonic diapause of *Bombyx mori* [5], [43], [44] and declines during the termination of diapause in adult linden tree bugs, *Pyrrhocoris apterus* [45]. The reduction of *Sodh* expression with the termination of diapause across insect orders and across embryonic, adult and now larval diapause indicates that declining *Sodh* expression is a general property of the termination of insect diapause and the initiation of post-diapause morphogenesis.

Sorbitol is generally described as a cryoprotectant for winter-diapausing insects [3] but many insects produce sorbitol as an end product of anaerobic metabolism [46]. High levels of *Sodh* expression during diapause and declining levels of expression at the termination of diapause may then relate as much to recovery from oxygen poor conditions as to a release from the general need for cryoprotection.

We propose that part of the diapause syndrome involves anticipatory preparation for potentially anoxic environments and that the changes in expression of *WsPepck* and *WsSodh* that we observed in *W. smithii* represent an adaptive physiological response to the transition from potentially hypoxic winter environments to normoxic spring environments when active development is resumed.

### Candidate Loci for the Photoperiodic Switch

Multiple loci are involved in the evolution of photoperiodic response in *W. smithii* as evidenced from both line-cross analysis [12], [47]–[49] and QTL mapping [29]. These studies all agree that the genetic architecture underlying this trait is highly complex with large dominance and epistatic effects. Therefore, a single locus will not emerge as *the* gene for photoperiodic response but rather an assortment of genes will be found that contribute in complex ways to photoperiodism. Of the genes showing differential expression in response to day length (Figure 3), *WsPpdrg1* (formerly *Ws13043* [2]) is both located under a QTL for the evolution of critical photoperiod [29] and shows positive association between the frequency of its northern allele and critical photoperiod after 25 generations of free recombination. Hence, three independent forward genetic approaches identify *WsPpdrg1* either as a candidate gene or as a gene tightly linked to genes involved in the photoperiodic switch.

*WsPpdrg1* is a member of a family of “cuticular proteins of low complexity” [31] whose homologs in *D. melanogaster* are included within a larger array of genes that are related to *retinin* which codes for a signaling protein that shows cornea-specific expression [50]. An intimate connection between cuticular proteins and response to shortening day lengths in the pea aphid has been implicated in the modification of neurotransmitters involved in photoperiodic signaling [36]. Hence, although the label “cuticle protein” implies a structural function, our results along with those in pea aphid heads indicate dynamic pleiotropic functions of these genes associated with photoperiodism.

### Conclusions

Photoperiodism provides a physiological switch mechanism that enables animals from rotifers to rodents to regulate the timing of important events in their seasonal life histories [24]. Despite this widespread use of photoperiod, the genes involved in the switch mechanism itself remain elusive in natural populations. Herein we take a forward genetic approach using microarrays and association analysis to identify candidate loci involved in the photoperiodic mechanism itself and in ensuing downstream events resulting in the termination of diapause in the pitcher-plant mosquito, *Wyeomyia smithii*. We have used a novel approach with the microarray: exposing diapausing larvae to a single day length that a southern population interprets as a long day and a northern population interprets as a short day. After six day's exposure to this day length, diapausing northern larvae have received zero long days and remain in diapause; by contrast, southern diapausing larvae have experienced six long days and at least 50% of the larvae have terminated diapause and are irrevocably committed to development. At this time, we are able to distinguish 30 genes that are differentially expressed in response to day length out of the approximately 4,000 independent genes on the array. These 30 genes fall into three general categories (1) genes with orthologs in *D. melanogaster* that are most directly interpreted as being involved either in post-diapause development or in the transition from an oxygen-poor winter (diapausing) environment to an oxygen-rich spring and summer (developing) environment, (2) orphan genes in *W. smithii* with no known orthologs or previous annotation, and (3) genes with clear orthologs in other dipterans, but no previous functional annotation. *WsPpdrg1* lies under a QTL for the evolution of photoperiodic response and is in linkage disequilibrium with photoperiodic response after 25 generations of free recombination. Hence three separate forward genetic approaches, including the work presented herein, point to *WsPpdrg1*, or a gene closely linked to *WsPpdrg1*, as a leading candidate gene for involvement in the photoperiodic switch.

Candidate gene studies, based on historical hypotheses of circadian involvement in photoperiodism [17] have greatly advanced our knowledge of the workings of the circadian clock, but they have done little to shed light on the mechanisms of the photoperiodic switch and its evolution in seasonal environments [14], [25], [51]. During these early stages of research into the molecular basis of the photoperiodic switch, forward genetic screens that are unbiased by historical inertia will increasingly be required to generate a strong foundation of candidate genes with which to pursue more functional studies [14], [52]. Only then, can we begin to dissect ecologically and socially relevant phenotypes related not only to photoperiodism and diapause, but also to understanding the potential for animal populations to respond to altered seasonal environments due to rapid climate change [16], [53].

## Methods

### Generation of the Hybrid Line for the cDNA Library

In order to maximize the coverage in our cDNA library of the among-population variation that exists within *Wyeomyia smithii*, we generated a hybrid line that includes both the nuclear and cytoplasmic genomic components of eight populations representing the latitudinal and altitudinal range of the species (Table 2). The hybrid line was created with a four-generation crossing design (Figure S2), that, in the first three generations, created eight lines that each had a single population's cytoplasmic component combined with all eight nuclear genomes. These eight lines were combined in the fourth generation that then contained the cytoplasmic and nuclear genetic variation present within all eight parental populations.

**Table 2:** Populations used in the creation of the eight-way hybrids along with their geographic location and critical photoperiods.

		Latitude	Longitude		Critical
Population^1^	State	(°N)	(°W)	Altitude (m)	Photoperiod
LI	AL	30.47	87.48	15	12.30^3^
WI	FL	30.08	84.98	10	12.14^2^
SH	NC	35.05	79.54	107	13.04^4^
GS	NC	34.11	78.31	20	12.72^2^
DB	NC	35.03	83.06	900	13.88^2^
HS	NC	35.06	83.20	1190	14.40^3^
KC	ME	46.22	68.33	365	15.43^2^
RY	WI	45.87	91.99	295	14.99^5^

1 Acronym for populations referred to in other papers from this lab. [28], [58].

2 Ref. [58].

3 Ref. [27].

4 Data from Population PM^1^, located 10 km west of Population SH.

5 Data from Population ML^1^, located 180 km east of Population RY.

To begin each generation of crosses, larvae were synchronized into diapause by rearing individuals from the day of oviposition under short-day (Light∶Dark = 8∶16) conditions for at least 30 days. At least 100 animals from each parental cross were then allowed to develop and mate under long-day (Light∶Dark = 18∶6), field-based conditions with a smooth, sine-wave thermoperiod that ranged from 13 to 29°C each day.

### Preparation of the cDNA Library

To allow for the greatest representation of variation in the cDNA library, RNA was collected from the eight-population hybrid line. Total RNA was extracted (TRIzol Reagent, Invitrogen) from three replicate samples of 30 III and 30 IV instar larval heads sampled at four times of day (1, 7, 13, 19 h after lights on) under both short-day (L∶D = 10∶14) and long-day (L∶D = 18∶06) conditions. These samples (all with a 260/280 ratio of 2.0±0.1) were pooled into a total RNA pool. One µg from this pooled RNA preparation was used to create a cDNA library using the Clontech SMART cDNA Library Construction Kit (BD Biosciences; Protocol #PT3000-1), following the manufacturer's instructions. Briefly, *Sfi*-I digested and purified cDNAs were directionally ligated and packaged into the Lamda TrplEx2 vector using Gigapack III Gold Packaging Extract (Stratagene). The library was amplified according the Kit's Instructions (BD Biosciences; Protocol #PT3000-1).

The phage library was converted to a plasmid library and transformed into competent *Escherichia coli* (BM25.8) (Clontech protocol PT3003-1). Individual colonies were isolated, and after overnight incubation, were used as templates for Polymerase Chain Reaction (PCR) amplification using the LD-amplimer primer pair from Clontech (forward: 5′ TCCGAGATCTGGACGAGC 3′; reverse: 5′TAATACGACTCACTATAGGG 3′) with a 5′ amine modification (IDT; /5AmMC12/). Sixteen reactions from each 96-well PCR plate were examined using 1.0% agarose gel electrophoresis to check product sizes. Insert sizes were between 0.25 kb and 3 kb, and a vast majority of these were single products.

PCR reactions were precipitated (2.5 volumes 95% EtOH, 1/10 volume 3 M NaOAc) and re-suspended in nanopure water. Reactions were then dried under vacuum and each reaction was re-suspended in 10 µl 3X SSC. The cDNA library was assessed for redundancy by sequencing 1000 random clones. Of the 1000 sequences, 43.2% were unique suggesting that the library has a redundancy of ∼60%. As the redundancy was low for a cDNA library, no normalization was performed. At the time of printing, the only elements on the array that were “known” were the 1,000 elements (10%) sequenced during the test of redundancy. Other elements of the library printed on the array were sequenced in light of the results of the gene expression work.

### Printing and Probing of Microarrays

The *W. smithii* microarrays, consisting of two replicates of 10,000 cDNA elements each, were printed on silylated aldehyde (CEL-1) glass slides at the Genomics Facility of the University of Oregon. The slides were printed, allowed to dry overnight, and then UV cross-linked at 6000 J. On the day of hybridization, slides were post-processed by immersing slides in a wash buffer (5X SSC, 0.1% SDS, 0.1 mg/mL BSA) at 42°C for 60 minutes, and washing the slides three times in 0.1X SSC at room temperature. The slides were then immersed in the blocking solution (2X SSC, 0.05% SDS, 2.5 mg/mL NaBH_4_) for 30 minutes at 42°C. After 3 washes in 0.1X SSC at room temperature, slides were dried by centrifugation at 800 rpm for 5 minutes.

Animals used in this study were from Ontario, Canada, and North Carolina, USA (Fig. 2), corresponding to localities DR and DB, respectively, in previous publications from our lab. These two populations are separated by >1,100 km. Total RNA was extracted (TRIzol Reagent, Invitrogen) from three replicates of 50 whole larvae for each sampling point (population and day treatments) within 20 minutes of 10 hours after lights-on. A reference sample containing an equimolar amount of each sample was used as one treatment on all arrays to facilitate comparisons among samples. For each array, 15 µg of each of two RNA samples (one experiment, one reference) was reverse transcribed to cDNA and labeled with Cy3 and Cy5 dUTP fluorescent dyes (PerkinElmer) using the Superscript cDNA Labeling System (Invitrogen) according to the manufacturer's protocol, but reducing the reagents by half. Unincorporated dyes were removed from the samples using a Qiagen PCR Purification Kit following the manufacturer's protocol. Samples were then dried completely under vacuum and re-suspended in 42 µL of hybridization buffer containing 50% Formamide, 3X SSC, 1% SDS, 5X Denhardt's solution (Sigma), and 0.8 mg/mL poly(A) (Sigma). Samples were then boiled at 100°C for 2 minutes, centrifuged, and added to the arrays. The arrays were covered by Lifterslips (Erie Scientific) and allowed to hybridize overnight in Corning microarray chambers submerged in a 42°C water bath.

Following overnight hybridization, arrays were washed at room temperature in 3 solutions containing: (1) 1X SSC and 0.015% SDS; (2) 0.2X SSC; and (3) 0.05X SSC. The microarrays were then dried by centrifugation at 800 rpm for 5 minutes. Microarrays were scanned using a GenePix 4000B scanner and software (Axon GenePix 6.0) and normalized to obtain a 635/532 nm ratio (of medians within arrays) between 0.90 and 1.1.

### Array Analysis

All data were analyzed using the LIMMA package for the statistical computing package R [54], [55] which implements empirical Bayesian methods for analyzing microarray data [56]. Hierarchical clustering of all elements showing a significant interaction effect was used to isolate those elements that showed differential expression between the one long-day response condition and the three short-day response conditions. The most ancestral node on the gene tree that included all elements showing this type of interaction defined the set of array elements that are included in this analysis. These elements were sequenced from the cDNA clone library, and homology was assigned by *blastx* search of the *Drosophila melanogaster*, *Aedes aegypti*, and *Anopheles gambiae* databases at NCBI. Gene names were assigned the names from the highest scoring blast hit from *D. melanogaster*, preceded by *Ws*. Where there were no significant blast results, genes were named *WsUnknown* followed by a number. Data for elements that represented the same transcript were pooled.

All gene-expression data are MIAME (Minimum Information About a Microarray Experiment) compliant and stored at the Gene Expression Omnibus (http://www.ncbi.nlm.nih.gov/geo/) under platform accession GPL9498.

### Real-Time PCR Confirmation of Microarray Data

In order to test the reliability of the microarray data, we used qRT-PCR to confirm the qualitative pattern of gene expression of four genes found in the microarray study–two that were up-regulated under long-day response treatments (*WsSodh-1* and *WsOho23B*), and two that were down-regulated under long-day response treatments (*WsPpdrg1* and *WsCpr65Az*). *Rp49* was used as the reference gene as in [18].

RNA was isolated using TRIzol as for the microarrays. For each treatment, qRT-PCR was performed on one randomly chosen RNA sample used in the array experiments, along with two independent replicates, for a total of three replicate RNA samples for each treatment. cDNA was synthesized following the Super-Script III Reverse Transcriptase protocol (Invitrogen), and real-time PCR was performed using the Sybr Green PCR Master Mix (Applied Biosystems) on an ABI PRISM 7900HT detection system (Applied Biosystems). The ABI software was used for calculating relative expression levels. Primers used for gene specific amplification can be found in Table S1.

Analysis of variance (ANOVA) was used to test for difference in gene expression among treatments for each of the four genes. Independent contrasts were used to test the explicit hypothesis that expression in the long-day response treatment (southern population, 6 long days) differed from the other three treatment [54], [57].

### Association Analysis

We established a north-south hybrid line by crossing a female from Florida (31°N) with a short critical photoperiod with a male from Alberta (58°N) with a long critical photoperiod. These localities are separated by >3,800 km and correspond to CR and AB, respectively, in previous papers from our lab. This cross generated 53 F_1_ offspring and thereafter was maintained with N>1,000 for 26 generations to allow free-recombination. In the F_26_ hybrid generation, we initiated diapause on short days and then, using incrementing day lengths [12], [29], [48], [49], determined the critical photoperiod of 862 individual larvae.

Because the F_26_ progeny of the cross is polymorphic for stage of diapause (individuals diapause as either III or IV instar larvae), both III and IV instar larvae were genotyped separately.

High and low critical photoperiod animals were isolated from each of the instar groups by taking individuals at ∼10^th^ percentile and ∼90^th^ percentile of the critical photoperiod distribution. Individuals of each of the four groups were genotyped for *WsPpdrg1* as in [29].

## Supporting Information

Figure S1 (A) Alignment of conserved RetininC superfamily domain in ppdrg1 and other members of the mosquito CPLCA family (Cornman and Willis 2009. Insect Molecular Biology 18:607–622). (B) Neighbor-joining tree of full sequence alignments of all CPLCA genes.
(0.17 MB PDF)

Figure S2 Crossing scheme for the generation of the eight-way hybrid line, designed to combine the nuclear and cytoplasmic genomes of eight populations representing the latitudinal and altitudinal range of Wyeomyia smithii (Table 2). The F1 generation consists of 16 reciprocal crosses that are then crossed to create 16 F2 lines that each include one of the parental cytoplasmic (represented by the colored lines) and 4 parental nuclear genomes. The F2 lines are crossed to create eight F3 lines, each including a single parental cytoplasmic genome and all eight parental nuclear genomes. Finally, the F3 lines are mass mated to form the eight-way hybrid line in the F4. In all crosses, >100 individuals of each sex were mated to create the next generation's line.
(0.22 MB PDF)

Table S1 Primers used for qRT-PCR. For Rp49 primers see Mathias et al. (2005, Journal of Insect Physiology 51:661–667).
(0.05 MB PDF)

Table S2 Association analysis of allele frequencies at WsPpdrg1 between high and low critical photoperiod individuals in an F26 generation from a cross of a northern individual with a southern individual. The lines and alleles are as described in Mathias et al. (2007, Genetics 176:391–402). The analysis is performed separately for III instar and IV instar larvae and is a Chi-squared test of independence with 1 degree of freedom using the Yates correction for continuity (Sokal and Rohlf. 1995, Biometry).
(0.11 MB PDF)

Text S1 Glossary of Terms
(0.03 MB DOC)

## References

[1] Danilevskii AS (1965). Photoperiodism and seasonal development of insects..

[2] Tauber MJ, Tauber CA, Masaki S (1986). Seasonal adaptations of insects..

[3] Leather SR, Walters KFA, Bale JS (1993). The ecology of insect overwintering..

[4] Koštál V (2006). Eco-physiological phases of insect diapause.. J Insect Physiol.

[5] Denlinger DL (2002). Regulation of diapause.. Annu Rev Entomol.

[6] Bradshaw WE, Zani PA, Holzapfel CM (2004). Adaptation to temperate climates.. Evolution.

[7] Taylor F (1980). Optimal switching to diapause in relation to the onset of winter.. Theor Popul Biol.

[8] Bradshaw WE (1976). Geography of photoperiodic response in diapausing mosquito.. Nature.

[9] Danks HV (1987). Insect dormancy: an ecological perspective..

[10] Bradshaw WE, Holzapfel CM (2001). Genetic shift in photoperiodic response correlated with global warming.. Proc Natl Acad Sci USA.

[11] Gomi T, Nagasaka M, Fukuda T, Hagihara H (2007). Shifting of the life cycle and life-history traits of the fall webworm in relation to climate change.. Entomol Exp Appl.

[12] Hard JJ, Bradshaw WE, Holzapfel CM (1993). The genetic basis of photoperiodism and evolutionary divergence among populations of the pitcher-plant mosquito, *Wyeomyia smithii*.. Am Nat.

[13] Hoy MA, Dingle H (1978). Variability in diapause attributes of insects and mites: some evolutionary and practical implications.. Evolution of Insect Migration and Diapause.

[14] Emerson KJ, Bradshaw WE, Holzapfel CM (2009). Complications of complexity: Integrating environmental, genetic and hormonal control of insect diapause.. Trends Genet.

[15] Saunders DS (2002). Insect clocks..

[16] Bradshaw WE, Holzapfel CM (2010). Light, time and the physiology of biotic response to rapid climate change.. Annu Rev Physiol In Press.

[17] Bünning E (1936). Die endonome Tagesrhythmik als Grundlage der photoperiodischen Reaktion.. Ber Dtsch Bot Ges.

[18] Mathias D, Jacky L, Bradshaw WE, Holzapfel CM (2005). Geographic and developmental variation in expression of the circadian rhythm gene, *timeless*, in the pitcher-plant mosquito, *Wyeomyia smithii*.. J Insect Physiol.

[19] Sauners DS (1990). The circadian basis of ovarian diapause regulation in *Drosophila melanogaster*: Is the *period* gene causally involved in photoperiodic time measurement?. J Biol Rhythms.

[20] Saunders DS, Henrich VC, Gilbert LI (1989). Induction of diapause in *Drosophila melanogaster*: Photoperiodic regulation and the impact of arrhythmic clock mutations on time measurement.. Proc Natl Acad Sci USA.

[21] Stehlík J, Závodská R, Shimada K, Šauman I, Koštál V (2008). Photoperiodic induction of diapause requires regulated transcription of *timeless* in the larval brain of *Chymomyza costata*.. J Biol Rhythms.

[22] Goto SG, Denlinger DL (2002). Short-day and long-day expression patterns of genes involved in the flesh fly clock mechanism: *period, timeless, cycle* and *cryptochrome*.. J Insect Physiol.

[23] Tauber E, Zordan M, Sandrelli F, Pegoraro M, Osterwalder N (2007). Natural selection favors a newly derived *timeless* allele in *Drosophila melanogaster*.. Science.

[24] Bradshaw WE, Holzapfel CM (2007). Evolution of animal photoperiodism.. Annu Rev Ecol Evol Syst.

[25] Bradshaw WE, Holzapfel CM (2007). Tantalizing *timeless*.. Science.

[26] Kyriacou CP, Peixoto AA, Sandrelli F, Costa R, Tauber E (2008). Clines in clock genes: fine-tuning circadian rhythms to the environment.. Trends Genet.

[27] Bradshaw WE, Lounibos LP (1977). Evolution of dormancy and its photoperiodic control in pitcher-plant mosquitoes.. Evolution.

[28] Emerson KJ, Letaw AD, Bradshaw WE, Holzapfel CM (2008). Extrinsic light:dark cycles, rather than endogenous circadian cycles, affect the photoperiodic timer in the pitcher-plant mosquito, *Wyeomyia smithii*.. J Comp Phys A.

[29] Mathias D, Jacky L, Bradshaw WE, Holzapfel CM (2007). Quantitative trait loci associated with photoperiodic response and stage of diapause in the pitcher-plant mosquito, *Wyeomyia smithii*.. Genetics.

[30] Benjamini Y, Hochberg Y (1995). Controlling the false discovery rate - a practical and powerful approach to multiple testing.. J Roy Stat Soc B Meth.

[31] Cornman RS, Willis JH (2009). Annotation and analysis of low-complexity protein families of *Anopheles gambiae* that are associated with cuticle.. Insect Mol Biol.

[32] Hynes RO, Zhao Q (2000). The evolution of cell adhesion.. J Cell Biol.

[33] Tan C, Stronach B, Perrimon N (2003). Roles of myosin phosphatase during *Drosophila* development.. Development.

[34] Le Trionnaire G, Jaubert S, Sabater-Munoz B, Benedetto A, Bonhomme J (2007). Seasonal photoperiodism regulates the expression of cuticular and signalling protein genes in the pea aphid.. Insect Biochem Mol Biol.

[35] Le Trionnaire G, Francis F, Jaubert-Possamai S, Bonhomme J, De Pauw E (2009). Transcriptomic and proteomic analyses of seasonal photoperiodism in the pea aphid.. BMC Genomics.

[36] Török I, Herrmann-Horle D, Kiss I, Tick G, Speer G (1999). Down-regulation of RpS21, a putative translation initiation factor interacting with P40, produces viable minute imagos and larval lethality with overgrown hematopoietic organs and imaginal discs.. Mol Cell Biol.

[37] Verras M, Theodoraki MA, Mintzas AC (2004). Cloning, characterization, and developmental expression of the ribosomal protein S21 gene of the Mediterranean fruit fly *Ceratitis capitata*.. Arch Insect Biochem Physiol.

[38] Robich RM, Rinehart JP, Kitchen LJ, Denlinger DL (2007). Diapause-specific gene expression in the northern house mosquito, *Culex pipiens* L., identified by suppressive subtractive hybridization.. J Insect Physiol.

[39] Paterson CG (1971). Overwintering ecology of the aquatic fauna associated with the pitcher plant *Sarracenia purpurea* L.. Can J Zool.

[40] Pratt CW, Cornely K (2004). Essential biochemistry..

[41] Feala JD, Coquin L, McCulloch AD, Paternostro G (2007). Flexibility in energy metabolism supports hypoxia tolerance in *Drosophila* flight muscle: metabolomic and computational systems analysis.. Mol Syst Biol.

[42] Luque T, Hjelmqvist L, Marfany G, Danielsson O, El-Ahmad M (1998). Sorbitol dehydrogenase of *Drosophila*: gene, protein, and expression data show a two gene system.. J Biol Chem.

[43] Niimi T, Yamashita O, Yaginuma T (1993). Developmental profile of the gene expression of a *Bombyx* homolog of mammalian sorbitol dehydrogenase during embryogenesis in non-diapause eggs.. Comp Biochem Physiol B Comp Biochem.

[44] Niimi T, Yamashita O, Yaginuma T (1993). A cold inducible *Bombyix* gene encoding a protein similar to mammalian sorbitol dehydrogenase - yolk nuclei dependent gene expression in diapause eggs.. Eur J Biochem.

[45] Koštál V, Tollarova M, Dolezel D (2008). Dynamism in physiology and gene diapause in a heteropteran transcription during reproductive diapause in a heteropteran bug, *Pyrrhocoris apterus*.. J Insect Physiol.

[46] Hoback WW, Stanley DW (2001). Insects in hypoxia.. J Insect Physiol.

[47] Bradshaw WE, Haggerty BP, Holzapfel CM (2005). Epistasis underlying a fitness trait within a natural population of the pitcher-plant mosquito, *Wyeomyia smithii*.. Genetics.

[48] Lair KP, Bradshaw WE, Holzapfel CM (1997). Evolutionary divergence of the genetic architecture underlying photoperiodism in the pitcher-plant mosquito, *Wyeomyia smithii*.. Genetics.

[49] Mathias D, Reed LK, Bradshaw WE, Holzapfel CM (2006). Evolutionary divergence of circadian and photoperiodic phenotypes in the pitcher-plant mosquito, *Wyeomyia smithii*.. J Biol Rhythms.

[50] Kim E, Choi Y, Lee S, Seo Y, Yoon J (2008). Characterization of the *Drosophila melanogaster retinin* gene encoding a cornea-specific protein.. Insect Mol Biol.

[51] Emerson KJ, Dake SJ, Bradshaw WE, Holzapfel CM (2009). Evolution of photoperiodic time measurement is independent of the circadian clock in the pitcher-plant mosquito, *Wyeomyia smithii*.. J Comp Phys A.

[52] Tauber E, Kyriacou CP (2008). Genomic approaches for studying biological clocks.. Funct Ecol.

[53] Bradshaw WE, Holzapfel CM (2008). Genetic response to rapid climate change: it's seasonal timing that matters.. Mol Ecol.

[54] R Development Core Team (2007). R: A language and environment for statistical computing..

[55] Smyth GK, Gentleman R, Carey V, Dudoit S, Irizarry R, Huber W (2005). Limma: linear models for microarray data.. Bioinformatics and computational biology solutions using R and Bioconductor.

[56] Smyth GK (2004). Linear models and empirical Bayes methods for assessing differential expression in microarray experiments.. Stat App Gen Mol Biol.

[57] Sokal RR, Rohlf FJ (1995). Biometry..

[58] Bradshaw WE, Quebodeaux MC, Holzapfel CM (2003). Circadian rhythmicity and photoperiodism in the pitcher-plant mosquito: adaptive response to the photic environment or correlated response to the seasonal environment?. Am Nat.

