# Supplementary material for: Microarrays Reveal Early Transcriptional Events during the Termination of Larval Diapause in Natural Populations of the Mosquito, Wyeomyia smithii
Source: PLoS One. 2010 Mar 5;5(3):e9574. doi: 10.1371/journal.pone.0009574 (PMC2832704; doi:10.1371/journal.pone.0009574)
Supplement: Figure S1 — (A) Alignment of conserved RetininC superfamily domain in ppdrg1 and other members of the mosquito CPLCA family (Cornman and Willis 2009. Insect Molecular Biology 18:607–622). (B) Neighbor-joining tree of full sequence alignments of all CPLCA genes. (0.17 MB PDF) [file pone.0009574.s001.pdf]

Figure S1

A

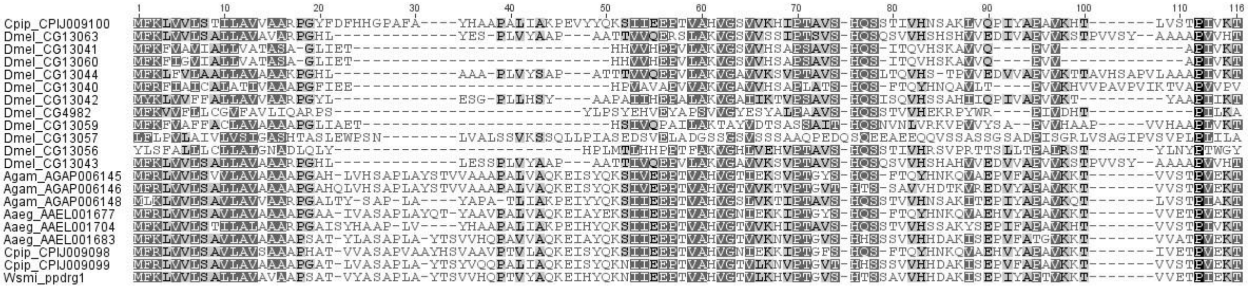

B

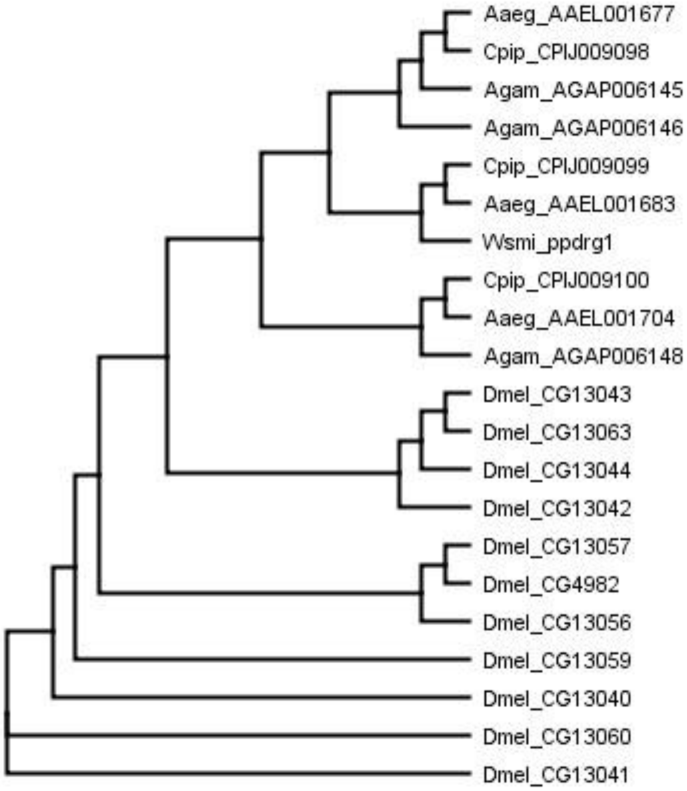

Figure S1. (A) Alignment of conserved RetininC superfamily domain in *ppdrg1* and other members of the mosquito CPLCA family (Cornman and Willis 2009. Insect Molecular Biology 18:607-622). (B) Neighbor-joining tree of full sequence alignments of all CPLCA genes.
