# Supplementary material for: Microarrays Reveal Early Transcriptional Events during the Termination of Larval Diapause in Natural Populations of the Mosquito, Wyeomyia smithii
Source: PLoS One. 2010 Mar 5;5(3):e9574. doi: 10.1371/journal.pone.0009574 (PMC2832704; doi:10.1371/journal.pone.0009574)
Supplement: Figure S2 — Crossing scheme for the generation of the eight-way hybrid line, designed to combine the nuclear and cytoplasmic genomes of eight populations representing the latitudinal and altitudinal range of Wyeomyia smithii (Table 2). The F1 generation consists of 16 reciprocal crosses that are then crossed to create 16 F2 lines that each include one of the parental cytoplasmic (represented by the colored lines) and 4 parental nuclear genomes. The F2 lines are crossed to create eight F3 lines, each including a single parental cytoplasmic genome and all eight parental nuclear genomes. Finally, the F3 lines are mass mated to form the eight-way hybrid line in the F4. In all crosses, >100 individuals of each sex were mated to create the next generation's line. (0.22 MB PDF) [file pone.0009574.s002.pdf]

Figure S2

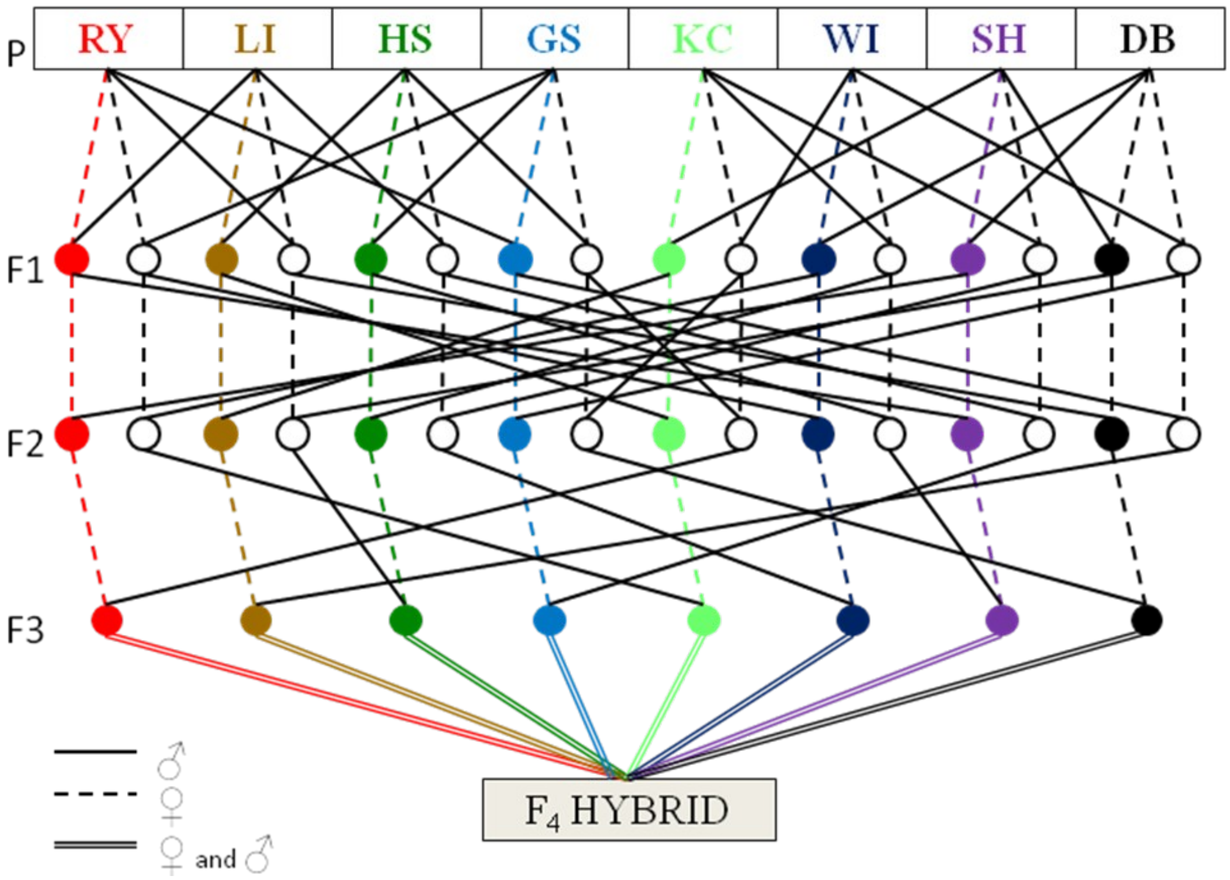

Figure S2. Crossing scheme for the generation of the eight-way hybrid line, designed to combine the nuclear and cytoplasmic genomes of eight populations representing the latitudinal and altitudinal range of *Wyeomyia smithii* (Table 2). The F_1 generation consists of 16 reciprocal crosses that are then crossed to create 16 F_2 lines that each include one of the parental cytoplasmic (represented by the colored lines) and 4 parental nuclear genomes. The F_2 lines are crossed to create eight F_3 lines, each including a single parental cytoplasmic genome and all eight parental nuclear genomes. Finally, the F_3 lines are mass mated to form the eight-way hybrid line in the F_4. In all crosses, > 100 individuals of each sex were mated to create the next generation's line.
