# Supplementary material for: Microarrays Reveal Early Transcriptional Events during the Termination of Larval Diapause in Natural Populations of the Mosquito, Wyeomyia smithii
Source: PLoS One. 2010 Mar 5;5(3):e9574. doi: 10.1371/journal.pone.0009574 (PMC2832704; doi:10.1371/journal.pone.0009574)
Supplement: Table S1 — Primers used for qRT-PCR. For Rp49 primers see Mathias et al. (2005, Journal of Insect Physiology 51:661–667). (0.05 MB PDF) [file pone.0009574.s003.pdf]

Table S1. Primers used for qRT-PCR. For *Rp49* primers see Mathias et al. (2005, Journal of Insect Physiology 51:661 – 667).

Gene	Direction	Primer Sequence	Amplicon Length
WsSodh-1	F	CCCAGCAGCGGTGGAGGTTG	100
	R	CGACGCGGAAGGCTTCGTGA	
WsOho23B	F	CACGCCAAGGATCATGCCTCCA	98
	R	ACCGCAGATAGCGTGCACCTTG	
WsPpdrg1	F	GCTGCCGCTCCGGCTTACAG	99
	R	CGGCGTAGCTCAGAGCAGGG	
WsCpr65Az	F	CGGGGAGTACGCTGCTCTCT	101
	R	GCGCCAACACCAGCACGTAT	
