# Supplementary material for: Microarrays Reveal Early Transcriptional Events during the Termination of Larval Diapause in Natural Populations of the Mosquito, Wyeomyia smithii
Source: PLoS One. 2010 Mar 5;5(3):e9574. doi: 10.1371/journal.pone.0009574 (PMC2832704; doi:10.1371/journal.pone.0009574)
Supplement: Table S2 — Association analysis of allele frequencies at WsPpdrg1 between high and low critical photoperiod individuals in an F26 generation from a cross of a northern individual with a southern individual. The lines and alleles are as described in Mathias et al. (2007, Genetics 176:391–402). The analysis is performed separately for III instar and IV instar larvae and is a Chi-squared test of independence with 1 degree of freedom using the Yates correction for continuity (Sokal and Rohlf. 1995, Biometry). (0.11 MB PDF) [file pone.0009574.s004.pdf]

Table S2. Association analysis of allele frequencies at *WsPpdrg1* between high and low critical photoperiod individuals in an F26 generation from a cross of a northern individual with a southern individual.  The lines and alleles are as described in Mathias et al. (2007, Genetics 176:391 – 402).  The analysis is performed separately for III instar and IV instar larvae and is a Chi-squared test of independence with 1 degree of freedom using the Yates correction for continuity (Sokal and Rohlf. 1995, Biometry).

| | Short CPP | | Long CPP | | | |
|---|---|---|---|---|---|---|
| Stage of Diapause | Southern Allele | Northern Allele | Southern Allele | Northern Allele | $\chi^2$ | P |
| III | 28 | 4 | 11 | 13 | 9.38 | 0.002 |
| IV | 30 | 0 | 21 | 11 | 10.30 | 0.001 |
