# Supplementary material for: Microarrays Reveal Early Transcriptional Events during the Termination of Larval Diapause in Natural Populations of the Mosquito, Wyeomyia smithii
Source: PLoS One. 2010 Mar 5;5(3):e9574. doi: 10.1371/journal.pone.0009574 (PMC2832704; doi:10.1371/journal.pone.0009574)
Supplement: Text S1 — Glossary of Terms (0.03 MB DOC) [file pone.0009574.s005.doc]

Glossary of terms used in the text:

**Critical photoperiod:** Population-level phenotype: the day length stimulating 50% development

and 50% dormancy in a sample population; the daylength at the inflection point of a

photoperiodic response curve (Bradshaw, W. E. and L. P. Lounibos. 1977. Evolution **31**(3): 546-567). Individual-level phenotype: the day length at which a diapausing individual terminates diapause in response to increasing day length (Mathias et al. 2007. Genetics **176**(1): 391-402).

**Diapause:** Dormancy in arthropods, fundamentally characterized by a cessation of development whether in embryonic, larval, pupal or adult (reproductive) stages. Diapause is a hormonally mediated developmental arrest that is anticipatory in nature and therefore generally is initiated in advance of seasonal exigencies.

**Forward genetics:** Forward genetic approaches seek to identify the genetic basis of a phenotype, that is, to identify mutations or naturally segregating alleles that produce a certain phenotype. Examples of this approach include fine-scale QTL mapping, expression microarrays, and association analysis. This approach contrasts with reverse genetics, which determines the phenotype that results from mutating a given gene.

**Photoperiodic switch:** The physiological mechanism that underlies the ability to use day length to cue seasonal events. The photoperiodic switch includes the **recognition** of day length as long or short by the photoperiodic timer, **integration** by the photoperiodic counter, and the **execution** of downstream events through the neuroendocrine system.

**Photoperiodism:** The ability to use day or night length to anticipate and prepare for seasonal change.

**QTL:** Quantitative trait loci, regions of an organism’s genome associated or correlated with phenotypic variation of a particular trait.
